# Genomic and phenotypic selection indices for decreased birth weight, shortening fattening period, and regulating total weight gain in beef cattle

**DOI:** 10.5713/ab.24.0171

**Published:** 2024-08-23

**Authors:** Kenji Togashi, Atsushi Ogino, Toshio Watanabe, Masakazu Shinomiya, Masashi Kinukawa, Kazuhito Kurogi, Masanobu Nurimoto

**Affiliations:** 1 Livestock Improvement Association of Japan, Maebashi, Gunma, 371-0121, Japan (Retired); 2 Livestock Improvement Association of Japan, Maebashi, Gunma, 371-0121, Japan; 3 Livestock Improvement Association of Japan, Koto-ku, Tokyo, 135-0041, Japan

**Keywords:** Birth Weight, Early Maturation, Growth Curve, Inbreeding, Index, Random Regression

## Abstract

**Objective:**

This study aimed to develop genomic and phenotypic indices for beef cattle selection that afford progeny with reduced birth weight and fattening period. The indices should also allow regulating total weight gain during the fattening period and avoid marked increases of inbreeding.

**Methods:**

Whether addition of constraint on total weight gain to constraints on body weight gain at up to four specific time points during the fattening process was effective for the weight gain until the end of the fattening period was examined in two selection indices with the selection trait being a phenotypic or a genomic breeding value random regression coefficient.

**Results:**

Both indices afforded cattle with desired weight gains at specific time points and desired total weight gains. One cycle of index-based selection made it possible to shorten the fattening period two weeks compared with that before selection while maintaining the same final fattening weight as before selection. The impact of constraining total weight gain was smallest when the number of weight gain constraints at specific time points was three or four. However, constraining total weight gain was necessary to avoid poor total weight gain when the number of weight gain constraints at specific time points was only one or two.

**Conclusion:**

The developed indices make it possible to regulate total weight gain during the fattening process and to achieve desired weight gains at specific time points. Importantly, the indices bring about genetic improvement without an excessive increase of inbreeding. Thus, we expect that these indices will contribute to sustainable genetic improvement in cattle while maintaining genetic diversity.

## INTRODUCTION

When breeding cattle, there is often genetic antagonism between the goal of producing food-animal progeny that show rapid, efficient, and early growth and the desire for small, low-maintenance parental strains [1–3]. However, increasing the growth characteristics of progeny while decreasing the size of parental strains risks increasing the incidence of dystocia (abnormal or difficult birth). Indeed, when selecting cattle for body weight, expected responses include a slightly greater increase in body weight and degree of maturity at age of selection but also substantial increases in body weight at all other ages, including at birth, due to positive genetic correlations between body weights during growth [4]. Therefore, progeny that show low birth weights are also desirable.

Previously, we used a random regression (RR) model to develop a point-gain index that can be used to select cattle that exhibit desired body weights at specific time points within the fattening period [5]. RR models have been applied for the genetic evaluation of longitudinal data such as growth, lactation, and egg production curves [6–10]. In particular, RR models have been applied to analyze the entire fattening process [11–14]. However, even though desired genetic gains at targeted times might be realized, any number of growth curves could be applied to achieve the same results, especially when the time points of interest are far apart. On the other hand, major factors influencing the rates of inbreeding were considered to be the number of parents and the selection intensity [15]. Therefore, we should select for the growth curves that afford the desired gains with the minimum amount of intensity of selection to minimize inbreeding, which will in turn increase the likelihood of achieving the selection goal.

In our previous study, we named the index that can achieve the desired weight gain at the target point through the fattening process as the point-gain index. We applied four constraints to our point-gain index at 0, 42, 86, and 130 weeks of age as the boundary ages that roughly divide the fattening process into three periods. We showed that the desired weight gain at each constrained time point was equal to that expected in cattle selected by using the developed index [5]. Importantly, to ensure that these values matched, we found that the number of constraints applied to the index needed to be less than or equal to the order of the RR coefficient and constant [5]. Ensuring that the number of constraints was within this limit reduced the need for increased intensity of selection and therefore reduced the risk of increased inbreeding. However, as the number of constraints is reduced, the applicability of the index across the entire fattening period decreases to only the time periods around the specified time points. Thus, a constraint is needed that increases the influence of the index on body weight during the entire fattening period.

Our previous point-gain index was based on RR coefficients of genomically enhanced breeding value (GEBV) [5], which are not always available. However, it is relatively easy to collect phenotypic values as an alternative to GEBVs. Thus, a selection index that uses phenotypic Legendre RR coefficients would be of value to cattle breeders.

Here, we developed two selection indices—one genomic and one phenotypic. The genomic selection index was based on our previously reported index [5]. The phenotypic selection index is newly reported. Both indices afforded progeny with decreased birth weight and increased postnatal growth. In addition to examining the effects of applying constraints on body weight gain at specific time points during the fattening period, we also examined the effects of applying a constraint on total body weight gain during the fattening period. Thus, we report here two selection indices that balance decreased birth weight with increased postpartum growth which leads to shortening the fattening period and afford breeders control of weight gain during the entire fattening period.

## MATERIALS AND METHODS

We developed the two point-gain indices—a phenotypic index (I_p_) and a genomic index (I_GEBV_)—by using a RR model based on Legendre polynomials. Both indices were developed to achieve desired weight gains at specific points during growth and a desired total weight gain by the end of the fattening period, which here was defined as the period from birth out to 130 weeks, with minimal intensity of selection. The mathematical bases of the two indices are discussed in the following subsections, followed by a numerical example to demonstrate the overall approach.

### Phenotypic index

To begin, a point-gain index with k orders of Legendre polynomials including a constant was defined as


$${\text{I}}_{\text{p}}=\Sigma_{j=0}^{k-1}\;b_{j}\;P_{j},$$

where *b_j_* is the index weight for the *jth* order of the Legendre polynomial coefficient, *P*_j_ is the *jth* order of the phenotypic Legendre RR coefficient including a constant, and *k*–1 is the maximum order of the Legendre polynomial function. In matrix notation, *I_p_* = ***b’P***, where ***b*** is a (*k*×1) column vector of index weights and ***P*** is a (*k*×1) column vector containing *P_j_* (*j* = 0,1,…,*k*–1) of phenotypic RR coefficients.

The desired genetic gain (Δ***G****_s_*) at specific time s during growth, and the desired total weight gain, are described using the following


(1) $$\begin{array}{l} \text{S}=\left[\begin{array}{ccccc} \phi_{0}(t_{1}) & \phi_{0}(t_{1}) & \phi_{2}(t_{1}) & \ldots & \phi_{k-1}(t_{1})\\ \phi_{0}(t_{2}) & \phi_{1}(t_{2}) & \phi_{2}(t_{2}) & \ldots & \phi_{k-1}(t_{2})\\ .. & .. & .. & \ldots & ..\\ \phi_{0}(t_{s}) & \phi_{1}(t_{s}) & \phi_{2}(t_{s}) & \ldots & \phi_{k-1}(t_{s})\\ \Sigma_{i=n,\;\;\;i\neq 1,2,\ldots ,s}^{m}\;\phi_{0}(t_{\text{i}}) & \Sigma_{i=n,\;\;\;i\neq 1,2,\ldots ,s}^{m}\;\phi_{1}(t_{\text{i}}) & \Sigma_{i=n,\;\;\;i\neq 1,2,\ldots ,s}^{m}\;\phi_{2}(t_{\text{i}}) & \ldots & \Sigma_{i=n,\;\;\;i\neq 1,2,\ldots ,s}^{m}\;\phi_{k-1}(t_{\text{i}}) \end{array}\right]\\ S\Delta \alpha_{L}=\Delta \mathrm{Gs}, \end{array}$$

and


$$\Delta G_{s}=[\begin{array}{ccccc} \Delta G_{t1} & \Delta G_{t2} & \ldots & \Delta G_{ts} & \Sigma_{i=n,i\neq 1,2,w,\ldots ,s}^{m}\;\Delta G_{ti} \end{array}],$$

where ***S*** is a (*s*+1)×*k* matrix; $\Sigma_{i=n,\;\;\;i\neq 1,2,\ldots ,s}^{m}\;\Delta G_{ti}$ is desired total weight gain (*i* = *n*,…*m*) excluding specific constrained time points (*i* ≠ *1*, *2*, .., *s*); Δ*G_ti_* is the desired genetic gain for the *ith* specific time point (*i* = *1*, *2*, …, *s*); *t_i_* is the age standardized for the *ith* time point (*i* = *n*,...,*m*); *s* is the total number of constraints at a particular time point; *ϕ_j_* (*t_i_*) is the jth order of the Legendre polynomial (*j* = *0,1*,.., *k*–1) evaluated at age *t_i_* standardized; and Δ***α****_L_* = (Δ*_αL_*__o__, Δ*_αL_*__1__)’, where Δ*_αL_*_*_i_*_ is the difference in the *ith* genetic Legendre RR coefficient (*α_L_*_*_i_*_) before and after selection (i.e., Δ*α_L_*_*_i_*_ = *α_L_*_*_i_*_ [after selection]– *α_L_*_*_i_*_ [before selection]). An (*s*+1) row vector in ***S*** is removed when the constraint on total weight gain was not applied.

The vector for the difference in the genetic Legendre RR coefficients after and before selection (i.e., Δ*α_L_*) can be described by using best linear unbiased prediction properties [16]:


$$\Delta \alpha_{L}=cov(\alpha_{L},I_{p})\frac{\bar{\iota }}{\sigma_{I_{p}}}=cov(\alpha_{L},P^{\prime })b\frac{\bar{\iota }}{\sigma_{I_{p}}}=G_{\alpha L}b\frac{\bar{\iota }}{\sigma_{I_{p}}},$$

where ***α****_L_* is the (*k*×1) vector of the true genetic Legendre RR coefficient, ***G****_αL_* is the (co)variance matrix of ***α****_L_*, *ῑ* is the intensity of selection, and *σ_I_*_*_p_*_ is the standard deviation of the phenotypic point-gain index. The value of *ῑ* required to achieve Δ***α****_L_* can be obtained by setting *ῑ* = *σ_I_*_*_p_*_. Therefore,


$$b={\left(G_{\alpha L}\right)}^{-1}\Delta \alpha_{L}.$$

and variance of *I_p_*, *i, e.*, is $\sigma_{I_{p}}^{2}$ described as


$$\begin{array}{l} \sigma_{I_{p}}^{2}=b^{\prime }V_{P_{\alpha L}}b=\Delta {\alpha_{L}}^{\prime }{\left(G_{\alpha L}\right)}^{-1}V_{P_{\alpha L}}{\left(G_{\alpha L}\right)}^{-1}\Delta \alpha_{L}\\ =\Delta {\alpha^{\prime }}_{L}{\left[G_{\alpha L}V_{p_{\alpha L}}^{-1}G_{\alpha L}\right]}^{-1}\Delta \alpha_{L}, \end{array}$$

where ***V****_P_*_*_αL_*_ is a (co)variance matrix of phenotypic Legendre RR coefficients.

We used a Lagrange multiplier to choose a vector, Δ***α****_L_*, such that the index constructed based on Δ***G****_s_* had the minimum variance, with the restriction that the vector of expected genetic gains at s specific time points during the fattening phase and desired total weight gain (Δ***G****_s_*) were equal to the vector of the pre-given desired and expected genetic gains (i.e., $\Delta G_{s}=[\begin{array}{ccccc} \Delta G_{t1} & \Delta G_{t2} & .. & \Delta G_{ts} & \Sigma_{i=n,\;\;\;i\neq 1,2,\ldots ,s}^{m}\;\Delta G_{ti} \end{array}]$). The function to be minimized is


$$f=\Delta {\alpha^{\prime }}_{L}{\left(G_{\alpha L}V_{p_{\alpha L}}^{-1}G_{\alpha L}\right)}^{-1}\Delta \alpha_{L}+\lambda^{\prime }(S\Delta \alpha_{L}-\Delta G_{s}),$$

where **λ′** = [λ_1_,λ_2_,…,λ*_s_*_+1_] is a vector of Lagrange multipliers. Setting the partial derivatives of function f with respect to Δ***α****_L_* equal to zero leads to


(2) $$\frac{\sigma f}{\sigma \Delta \alpha_{L}}=2{\left(G_{\alpha L}V_{p_{\alpha L}}^{-1}G_{\alpha L}\right)}^{-1}\Delta \alpha_{L}+S^{\prime }\lambda =0.$$

Setting the partial derivatives of function *f* with respect to **λ** equal to zero leads to


(3) $$\frac{\sigma f}{\delta \lambda }=S\Delta \alpha_{L}-\Delta G_{s}=0.$$

Equations (2) and (3) are then combined to produce


(4) $$\left[\begin{array}{cc} 2{\left[G_{\alpha L}V_{p_{\alpha L}}^{-1}G_{\alpha L}\right]}^{-1} & S^{\prime }\\ S & 0 \end{array}\right]\;\left[\begin{array}{c} \Delta \alpha_{L}\\ \lambda \end{array}\right]=\left[\begin{array}{c} 0\\ \Delta G_{s} \end{array}\right].$$

According to the principle of Lagrange multipliers, solution vector Δ***α****_L_* in equation (4) affords the minimum intensity of selection needed to satisfy the applied constraints such that the genetic gains expected when the selection index is used are equal to the pre-given desired genetic gains.

The inverse of the coefficient matrix of equation (4) is obtained through inversion by partitioning [17]. The solution to equation (4) is


(5) $$\Delta \alpha_{L}=G_{\alpha L}V_{p_{\alpha L}}^{-1}G_{\alpha L}S^{\prime }{\left(SG_{\alpha L}V_{p_{\alpha L}}^{-1}G_{\alpha L}\;S\right)}^{-1}\Delta G_{s}.$$

Conversely, Δ***α****_L_* in (5) has to satisfy the pre-specified gains as shown in (1) (i.e., ***SΔα****_L_* = Δ***G****_s_*), which is proven as


$$S\Delta \alpha_{L}=SG_{\alpha L}V_{p_{\alpha L}}^{-1}G_{\alpha L}S^{\prime }{\left(SG_{\alpha L}V_{p_{\alpha L}}^{-1}G_{\alpha L}\;S\right)}^{-1}\Delta G_{s}=\Delta G_{s}.$$

Index coefficients (***b***) for the selection index based on phenotypic Legendre RR coefficients are provided by


$$b={\left(G_{\alpha L}\right)}^{-1}\Delta \alpha_{L}.$$

Finally, the point-gain index based on phenotypic Legendre RR coefficients that would achieve the pre-specified gains at specific times with the least intensity of selection is calculated using


$${\text{I}}_{\text{p}}=P_{\alpha L}^{\prime }b=P_{\alpha L}^{\prime }[V_{p_{\alpha L}}^{-1}G_{\alpha L}\;S^{\prime }{\left(SG_{\alpha L}V_{p_{\alpha L}}^{-1}G_{\alpha L}\;S^{\prime }\right)}^{-1}\Delta G_{s}]$$

### Genomic point-gain index

The genomic point-gain index was defined as


$${\text{I}}_{\text{GEBV}}=\Sigma_{j=0}^{k-1}\;b_{j}\;GEBV_{j},$$

where *b_j_* is the index weight for the *jth* order of the GEBV Legendre RR coefficient, and *GEBV_j_* is the *jth* order of the GEBV Legendre RR coefficient including a constant. When total weight gain was constrained, the matrix (***S***) must include the (*s*+*1*)*th* row vector of (1), signifying the total weight gain. The remaining procedure is the same as we reported previously [5]. In short, the point-gain index based on the GEBV Legendre RR coefficients that would achieve the pre-specified gains at specific times and pre-given total weight gain during entire growth process with the least intensity of selection is calculated using


$${\text{I}}_{\text{GEBV}}=\mathrm{GEB}V_{\alpha L}^{\prime }b=\mathrm{GEB}V_{\alpha L}^{\prime }[S^{\prime }{\left(SV_{\mathrm{GEB}V_{\alpha L}}S^{\prime }\right)}^{-1}\Delta G_{s}],$$

with variance of the index, $\sigma_{I_{GEBV}}^{2}=\Delta \alpha_{L}^{\prime }{\left(V_{{\mathrm{GEBV}}_{\alpha L}}\right)}^{-1}\Delta \alpha_{L}$, where ***GEBV****_αL_* is the column vector of the GEBV for the Legendre RR coefficients and *V_GEBVαL_* is a (co)variance matrix of GEBV Legendre RR coefficients.

### Numerical example

We assumed a phenotypic covariance matrix of Legendre RR coefficients during the fattening process in Japanese Black steers [18,19] (Appendix 1). In addition, quartic Legendre polynomials [(*k*–1) = 4] were assumed, as done previously [5,13,20,21].

Japanese Black steers are slaughtered at around 30 months of age [22], so we fitted a growth curve out to 130 weeks. We assumed that the growth curve before selection was similar to that shown in Takeda et al [18], to which a Gompertz curve was fitted. However, we instead fitted a Legendre RR model to the curve to develop a selection index based on phenotypic RR coefficients. Growth curves were derived such that the birth weight was less than before selection for breeding and the desired final weight was achieved two weeks earlier than before selection (i.e., at 128 weeks of age) while also controlling weight gain during the entire fattening period.

Four conditions were examined whether addition of constraint on total weight was effective. These four conditions are due to differences in the method of setting specific constraints on the amount of weight gain during the fattening period. The constraints for four conditions were then

condition 1) birth weight (−2.5 kg)
condition 2) birth weight (−2.5 kg) and weight at 128 weeks (+4.6 kg)
condition 3) birth weight (−2.5 kg), weight at 66 weeks (+2.8 kg), and weight at 128 weeks (+4.6 kg)
condition 4) birth weight (−2.5 kg), weight at 43 weeks (+1.4 kg), weight at 87 weeks (+3.9 kg), and weight at 128 weeks (+4.6 kg).

Therefore within each condition, constraints were combined with or without a constraint on total weight gain, resulting in a total of eight constraint combinations.

The literature generally does not contain values for total weight gain out to 130 weeks for Japanese Black steers. However, values of yearly gain of carcass weight are published, which indicate a gain of 5 kg/yr at the final fattening weight [22,23]. Thus, the annual total weight gain (Δ*G_total gain_*) from birth through to 130 weeks of age was calculated as $\Delta G_{total\;gain}=\frac{\Sigma_{i=n,\;\;\;i\neq 1,2,\ldots ,s}^{m}\;\Delta G_{ti}}{\sigma_{{\text{G}}_{130}}^{2}}\Delta G_{133}$, excluding constraints for specific weight gains and specific time points (*i* = *1*, *2*, …, *s*), where $\sigma_{{\text{G}}_{130}}^{2}$ is the genetic variance of body weight at 130 weeks, *σ_G_*_*_i_*_,130__ is the genetic covariance of weights between the *ith* week of age and 130 weeks, and Δ***G*_130_** is annual weight gain at 130 weeks (assumed to be 5 kg). Similarly, weight gain at 43, 66, and 87 weeks of age was calculated as $\frac{\sigma_{G_{130},i}}{\sigma_{G_{130}}^{2}}\Delta G_{130}$, where *i* = 43, 66, or 87.

Weight gain at 128 weeks (+4.6 kg) was set so that the final weight at 130 weeks was achieved two weeks earlier compared with that before selection for breeding. This leads to shortening the fattening period. Growth curves were chosen such that body weight at birth was approximately 2.5 kg less than that before selection for all eight constraint combinations. Genetic (co)variance between *i* and *j* weeks of age was computed as *σ_G_*_*_i,j_*_ = ***ϕ****_i_****G****_αL_****ϕ****_j_****′***, where ***ϕ****_i_* is the vector of the Legendre polynomial (*j* = *0*,*1*, …, *k*–1) evaluated at age *t_i_* standardized and ***G****_αL_* was obtained from [5]. The reliability of *GEBV_j_* (*j* = *0*,*1*, ..., *k*–1) for the *jth* order of Legendre RR coefficients was assumed to be 0.7.

## RESULTS AND DISCUSSION

### Weight gains achieved using the developed selection indices with or without constraining total weight gain

We began by applying each of the combinations of constraints to the two indices (see Numerical example and Table 1). The body weights achieved by using the GEBV index are shown in Table 2, and those achieved by using the phenotypic index are shown in Table 3. For both indices, all of the body weights after selection were equal to the values computed by adding the desired weight gain to the body weight before selection. For example, in condition of 3 in Tables 2 and 3, specific times for desired gains are birth, 66, and 128 weeks of age with −2.5 kg, 2.8 kg, and 4.6 kg of desired gains, respectively. Body weights after selection at birth, 66, and 128 weeks of age were equal to the sum of body weights before selection and desired gains. Desired total weight gain during the fattening period, excluding given body weight gains at specific times, was equal to the achieved total gain after selection, that is, the difference in the body weights throughout the fattening period between after and before selection (i.e., body weight after selection – body weight before selection). Thus, the two indices afforded progeny with desired weight gains at specific weeks of age and a desired total weight gain until the end of the fattening period.

The intensities of selection needed to achieve the desired body weight gain with the two indices under the eight constraint conditions are shown in Table 4. For both indices, intensity of selection increased with increasing number of constraints applied. In addition, intensity of selection was higher when total weight gain was constrained. Overall, intensity of selection was higher for the phenotypic index than for the GEBV index, regardless of the number of constraints applied or whether total weight gain was constrained. Thus, the GEBV index afforded the same weight gains as did the phenotypic index but with less inbreeding. To compare the variation of phenotypic, genomic, and true RR coefficients, we calculated the variation of the entire 0, 1, 2, 3, and 4th-order Legendre RR coefficients (i.e., the sum of all the elements of the (co)variance matrix of Legendre RR coefficients) for the two indices. The values for the GEBV and phenotypic indices were 4,458.0 and 29,519.8, respectively. For comparison, the value for the true genetic Legendre RR coefficients was 6,389.5. Therefore, the ratio of the true genetic value to the phenotypic index value was 0.216, and the ratio of the GEBV value to the true genetic value was 0.7. Note that the value of 0.7 is the same as the reliability calculated for the GEBV RR coefficients (see Numerical example). Thus, the GEBV RR coefficients more closely matched the true genetic values than did the phenotypic RR coefficients, which explains the lower selection intensities obtained with that GEBV index than with the phenotypic index.

### Effects of constraining weight gain

**Figure 1:**
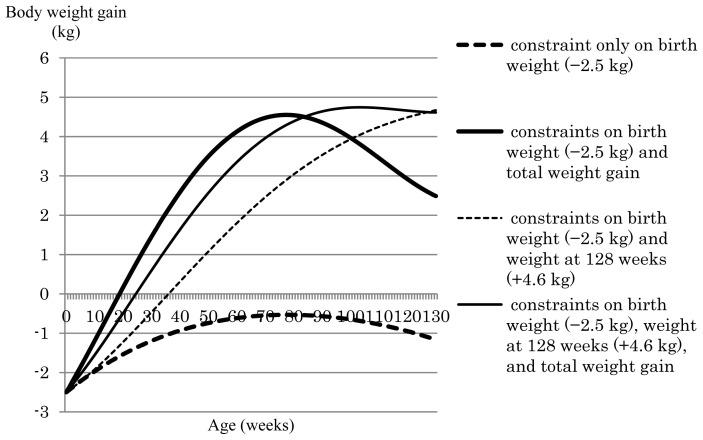
Growth curves afforded by the genomically enhanced breeding value–based index. Constraints were applied to birth weight, weight at 128 weeks, and total weight gain.

**Figure 2:**
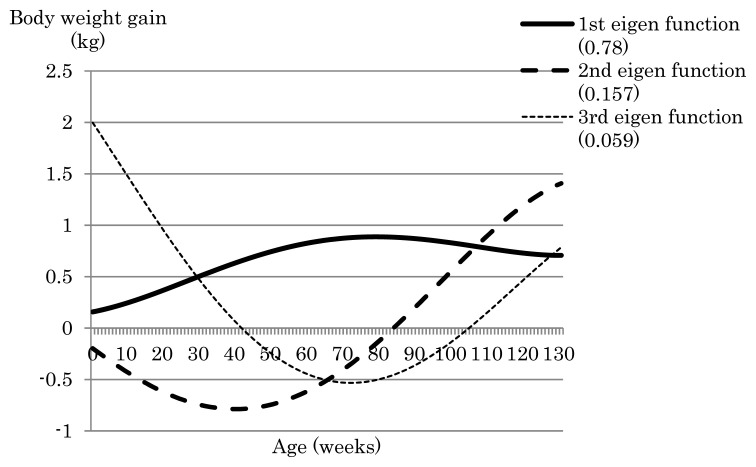
Eigenfunctions of the (co)variance of genomically enhanced breeding value for Legendre random regression coefficients. Values in parentheses indicate the proportion of the eigenfunction explaining the variance of the genomically enhanced breeding value over the trajectory.

Next, we examined the effects of constraining body weight gains at different time points with or without also constraining total weight gain. Figure 1 shows the weight gain curve over the trajectory obtained in the GEBV index with constraints placed on birth weight (−2.5 kg) or birth weight (−2.5 kg) and weight gain at 128 weeks (+4.6 kg) in relation to the presence or absence of restrictions on total weight gain. When the only constraint applied was to birth weight without constraining total weight gain, weight gain from birth to 130 weeks ranged from −2.5 kg to −0.53 kg, with the weight gain curve gradually increasing up to around 80 weeks after birth and decreasing thereafter. Thus, this single constraint on birth weight afforded a negative weight gain compared with before selection. The first to third eigenfunctions from birth to 130 weeks of age for the (co)variance matrix of the GEBV Legendre RR coefficients are shown in Figure 2. The first eigenfunction, which explained 78% of the variance over the trajectory, increased up to 80 weeks of age and decreased thereafter, which is consistent with the weight gain curve afforded by the GEBV index when the only constraint was applied to birth weight (Figure 1). When constraints were applied to birth weight (−2.5 kg) and total weight gain (+347.4 kg), the weight gain curve rapidly increased and showed a positive weight gain from around 20 weeks of age through to the end of the fattening period (Figure 1). Thus, the addition of a constraint on total weight gain was effective to avoid negative weight gain during the fattening process. When the number of constraints at specific time points was increased to two (birth weight [−2.5 kg] + weight at 128 weeks [+4.6 kg]) and the constraint on total weight gain was applied, the total weight gain was +342.4 kg, which matched the desired weight gain before selection (Table 2). In contrast, when the constraint on total weight gain was removed, the total weight gain was 222.9 kg (Table 2), which is a change of almost 120 kg between the conditions with and without the total weight gain constraint. Thus, constraining total weight gain was effective to produce a greater total weight gain when one or two constraints were applied to weight gain at specific time points during fattening.

We compared the difference in the achieved total weight gain after selection with and without a constraint on total weight gain when either one/two or three/four time-point constraints were applied (Tables 2 and 3). The difference in the achieved total weight gain after selection due to the presence or absence of a constraint on total weight gain was smaller when the number of constraints was three or four than when it was one or two, irrespective of the GEBV or phenotypic index (Tables 2 and 3). That is, when the number of time-point constraints was three or four, the achieved total weight gain from 0 to 130 weeks was largely unchanged irrespective of whether total weight gain was constrained. As already discussed, constraining total weight gain made it necessary to increase the intensity of selection and the rate of inbreeding [15]. Thus, the impact of constraining total weight gain was smallest when the number of time-point constraints was three or four.

**Figure 3:**
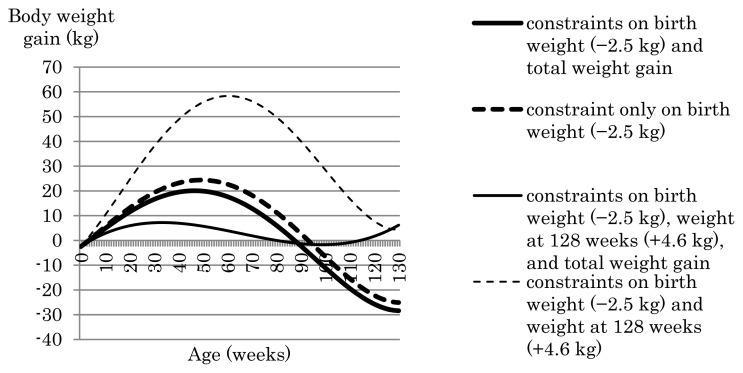
Growth curves afforded by the phenotypic index. Constraints were applied to birth weight, weight at 128 weeks, and total weight gain.

**Figure 4:**
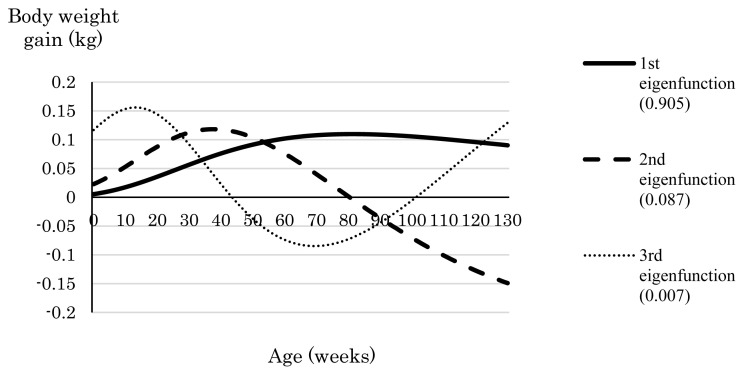
Eigenfunctions of the (co)variance of phenotypic Legendre random regression coefficients. Values in parentheses indicate the proportion of the eigenfunction explaining the variance of the phenotypic value over the trajectory.

**Figure 5:**
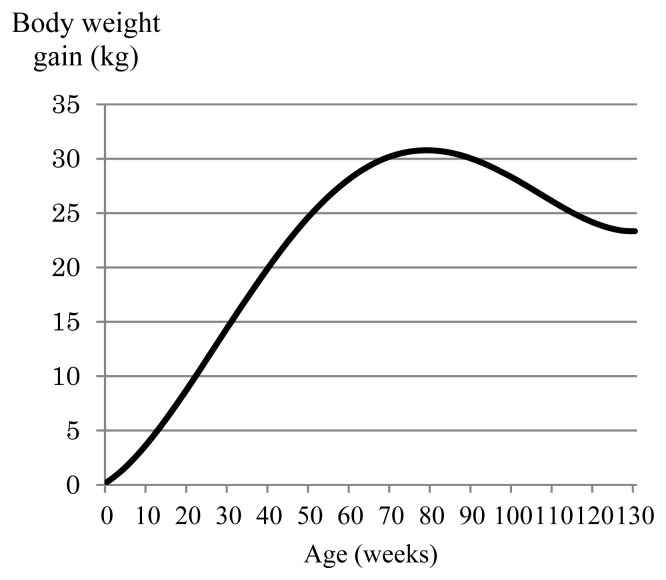
Selection response calculated using the 1st eigenfunction of the phenotypic Legendre random regression matrix when intensity of selection was set at 1.0.

Figure 3 shows the weight gain curve over the trajectory obtained in the phenotypic index with constraints placed on birth weight (−2.5 kg) or birth weight (−2.5 kg) and weight gain at 128 weeks (+4.6 kg) in relation to the presence or absence of a constraint on total weight gain. When the only constraint applied was to birth weight, the weight gain curve showed a convex shape irrespective of whether total weight gain was also constrained (Figure 3). A similarly shaped weight gain curve was obtained when constraints were applied to birth weight and weight at 128 weeks but not to total weight gain (Figure 3). The eigenfunctions of the (co)variance matrix of the phenotypic Legendre RR coefficients are shown in Figure 4. The first eigenfunction, which explained 90.5% of the variance over the trajectory, showed a convex shape, which is consistent with the shapes of the observed weight gain curves. The first eigenfunction of the (co)variance matrix of the phenotypic Legendre RR coefficient does not always reflect the genetic trend; therefore, we plotted the selection response due to the first eigenfunction when intensity of selection was set at 1.0 so that shape of the selection response can be compared (Figure 5; Appendix 2). The selection response also showed a convex shape. Thus, we concluded that the trend of weight gain afforded by the phenotypic index when the number of constraints was small (i.e., ≤2) appears to reflect the first eigenfunction of the (co)variance matrix of the phenotypic Legendre RR coefficients.

Taken together with GEBV index as well as phenotypic index, when the number of constraints on body weight gain at specific time points and on total weight gain was small (i.e., ≤2), the weight gain afforded by the index appeared to be greatly influenced by the eigenfunction of the (co)variance matrix of the GEBV or phenotypic RR coefficients in the population. This is likely because the characteristics of these parameters in relation to growth are more likely to be exhibited when the selection index has little constraints on weight gain during the growth process. The index may reveal the potential weight gain that can be achieved in a population with the given parameters. Therefore, we need to carefully investigate the weight gain achieved with the index when the number of constraints applied is small (i.e., ≤2).

When the phenotypic index was used and constraints were placed on birth weight and weight at 128 weeks but not on total weight gain, the achieved total weight gain was 4,369.2 kg (Table 3). The total gain value is excluding weight gain by the weeks of age at birth and 128 weeks of age. In contrast, when the total weight gain constraint was added, the achieved total weight gain was 342.4 kg, which matched the desired total weight gain before selection (Table 3).

**Figure 6:**
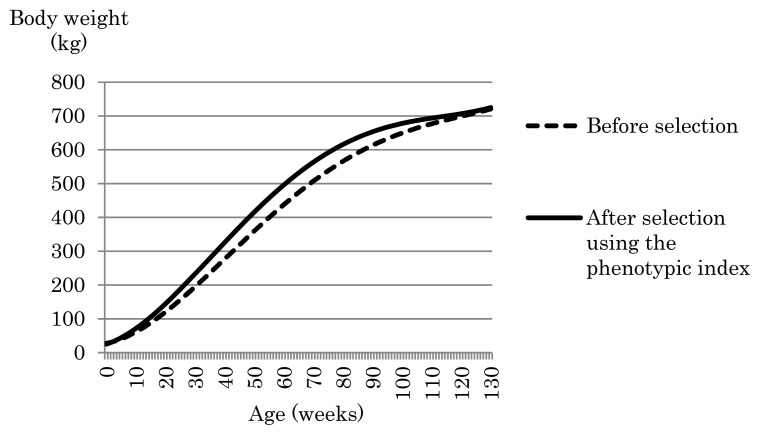
Growth curves of body weight obtained before and after selection using the phenotypic index. Constraints were applied to the index for birth weight (−2.5 kg) and weight at 128 weeks (+4.6 kg).

**Figure 7:**
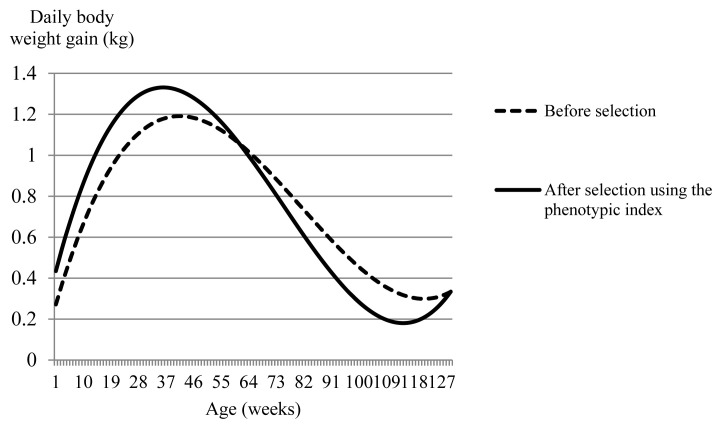
Growth curves of daily body weight gain obtained before and after selection using the phenotypic index. Constraints were applied to the index for birth weight (−2.5 kg) and weight at 128 weeks (+4.6 kg).

To examine the effects of the phenotypic index that has constraints on birth weight and weight at 128 weeks but not on total weight gain on growth during fattening process, we plotted body weight and daily weight gain by age afforded by the phenotypic index, and compared the body weight and daily weight gain curves with those obtained before selection, in Figures 6 and 7, respectively. In addition, daily gain at i weeks of age was computed by dividing the difference between body weight at i weeks of age and that at (i – 1) weeks of age by seven. After selection using the phenotypic index, body weight was smaller from birth through to the second week of age compared with that before selection. However, from the third week onward, body weight was greater after selection with the index than before selection. In particular, the index afforded a marked increase exceeding 30 kg in body weight gain from around 30 weeks of age through to around 90 weeks of age compared with that before selection. Daily weight gain from birth through to 60 weeks of age was greater after selection using the phenotypic index than that before selection (Figure 7). Particularly, the tendency for daily weight gain after selection to be greater than before selection was greatest around 16 weeks of age. The daily gain at 16 weeks of age after and before selection was 1.07 kg and 0.86 kg, respectively. However, the decline in the daily gain from 60 weeks of age through to the end of fattening was steeper after selection than before selection. As a result, as age progressed, the tendency for post-selection weight to exceed pre-selection weight decreased, while body weight after selection using the phenotypic index was still greater than before selection even after 60 weeks of age (Figure 6). This was confirmed by comparing the difference in the genetic RR coefficients corresponding to constant after and before selection (i.e., Δ*α_L_*_*_constant_*_ = *α_L_*_*_constant, after selection_*_ – *α_L_*_*_constant, before selection_*_). Figure 3 has four kinds of phenotypic indices based on different constraints, i) constraint only on birth weight (−2.5 kg), ii) constraints on birth weight (−2.5 kg) and total weight gain, iii) constraints on birth weight (−2.5 kg) and weight at 128 weeks (+4.6 kg), and iv) constraints on birth weight (−2.5 kg), weight at 128 weeks (+4.6 kg), and total weight gain. The difference in the genetic RR coefficients corresponding to constant after and before selection for i), ii), iii), and iv) was 9.1 kg, 3.9 kg, 47.5 kg, and 3.7 kg, respectively. The phenotypic index iii) with constraints on birth weight (−2.5 kg) and weight at 128 weeks (+4.6 kg) has increased constant part in RR body weight curve, i.e., body weight itself, during growth much greater than the other phenotypic indices. As a result, the increase in body weight due to the phenotypic index iii) appears to have contributed to the large value increase in the total weight gain during the fattening process (4,369.2 kg).

The maximum daily gain of +1.33 kg occurred at 36 weeks of age after selection and the maximum of +1.18 kg occurred at 41 weeks before selection (Figure 7). Thus, the daily weight gain after selection peaked earlier than that before selection, and the rate of decrease in daily gain after the peak was steeper with the index than before selection. This may suggest a shift towards a precocious growth curve associated with selection. The maximum average daily gain for late maturing pigs reached a higher and 10-day later peak than the early maturing pigs [24]. This study was conducted in steers. Genetic improvements in the growth of bulls cannot but influence the growth of cows as well. Consequently, the selection that will make peak in daily gain earlier than before selection may lead to a precocious growth curve for cows as well as steers. A Japanese Black heifer with a faster rate of maturing was suggested to show a higher conception rate [25]. The lowest age at first calving is for Aberdeen Angus, which is considered an early maturing breed, and the highest is for Charolais, which is considered a late-maturing breed [26]. Therefore, it will be necessary to clarify the effects of selection for an early maturing growth curve not only in steers but also in cows, especially from the perspective of how it affects the reproduction of cows. Genetic improvements that increase the size of bulls also work in the same direction for cows, which result in higher feed requirements, larger cow size, and higher feeding costs [27]. Crossbreeding may solve this problem; however, genetic improvement in Japanese black breeds is carried out within the breed. Thus, the use of an index-based selection approach to reduce birth weight and reach final fattening weight earlier than before selection might provide a means of obtaining improvements that allow for increased growth in bulls and earlier maturation and smaller size in females.

In the present study, total weight gain was calculated by using published data for annual improvement in body weight at the end of fattening [22,23]. Weight at the end of fattening is an important trait for beef cattle breeders because it is the final sales trait. Furthermore, the weight gain during the whole fattening process or the weight gain per time until the end of the fattening period is an important trait from the viewpoint of feed utilization efficiency (i.e., how much feed is needed for a given gain) and from the viewpoint of shortening the fattening period, which will ensure reducing the amount of feed or methane emissions. In particular, fattening of Japanese black breeds takes place over a long period of time until around 30 months of age. As the fattening period becomes longer, feed utilization efficiency decreases [28]. The indices developed here make it possible to reach the final fattening weight two weeks earlier than before selection after one cycle of selection. Further research is needed to examine by how much the fattening period can be shortened while maintaining the same final fattening weight and meat quality from the viewpoints of reducing the amount of feed or methane emissions and increasing feed efficiency. If the values of the degree of genetic improvement in weight gain over the entire growth period become available at a future time, it will be possible to assign a more accurate desired total weight gain value to the index than the value that was used in the present study.

Genotype by environment interaction occurs when performances of different genotypes are not equally affected by different environments [29]. The ability of living organisms (plants or animals) to alter the phenotype in response to changes in the environment is known as phenotypic plasticity or environmental sensitivity [30]. When the same genotypes develop different phenotypes in different environments, then there is genotype by environment interaction. High estimates of genetic correlation between environments (>0.80) indicate little or no evidence of strong genotype by environment interactions [31]. Genotype can refer to a genotypic unit (breeds, crossbreds, individuals), but also to a genotypic value (GEBVs). Under climatic conditions, production systems, and markets different from those where candidate animals were evaluated, the genotype by environment interaction can cause a reduced efficiency of genetic improvement programs when genetic correlations between environments are low. Legendre regression coefficients corresponding to genetic parameters related to growth pattern are obtained ignoring genotype by environment interaction in this study. Therefore, a reduced efficiency of genetic improvement in the point-gain index due to genotype by environment interaction would not be caused when genetic correlations of the point-gain index between environments are high. Correlation of the point-gain index between environments might be served as an accuracy of the point-gain index between environments.

Sire effect was partitioned into two parts: constant effect unaffected by environments and interaction effect specific to each environment and responsible for genotype by environment interaction [32]. Provided that Legendre regression coefficients were partitioned into two parts (constant effect and interaction effect specific to each environment) by multi-trait model, accuracy of the point-gain index based on Legendre regression coefficients corresponding to a constant effect may serve as a criterion whether a unique point-gain index is needed for each environment. The accuracy of the point-gain index with Legendre regression coefficients based on constant effects can be obtained as the correlation between the point-gain index obtained from each environment and the point-gain index based on constant effects.

Genetic parameters for final weight of young bulls tested on pasture or in feedlots were analyzed [33]. When the selection intensity was kept the same for both the environments, the greatest direct responses for final weight were exhibited by the animals reared and selected in feedlots. When the selection intensity on pasture was higher than the selection intensity in feedlots, the responses to direct selection were similar for both the environments and correlated responses obtained in feedlots by selection on pasture were similar to the direct responses in feedlots. That is, the same weight gain was shown in both the environments and the interaction between genotype and environment was seemingly eliminated. Selection intensity played an important role in the study of genotype by environment interaction in beef cattle [33]. Selection intensity increased with decreasing reliability of GEBV for point-gain index to achieve the intended weight gains [5]. Therefore, increase in selection intensity might relieve a reduced efficiency of genetic improvement when reliability of GEBV decreases in line with genotype by environment interaction. Further research would be necessary to clarify the effect of genetic by environment interaction on the point-gain index.

The purpose of this study was not to apply RR curves to the fattening process [10–13], but rather to develop selection indices that make it possible to control weight gain during the entire fattening process and to achieve desired weight gains at specific time points. Since the developed indices can bring about genetic improvement with minimal increase of inbreeding, we expect that these indices will also contribute to sustainable genetic improvement while maintaining genetic diversity. Selection indices developed to control growth and achieve specific weight gains at specific ages might be easily extended for use in plants and fish.

## CONCLUSION

Here, we developed two selection indices based on GEBV or phenotypic Legendre RR coefficients that make it possible to achieve a desired total weight gain during the fattening period and specific weight gains at specific time points. When there were few constraints on specific weight gains at specific ages and a desired total weight gain, the selection intensity required to obtain a satisfactory growth curve was lower than when the number of constraints was large. In other words, when there are fewer age constraints, growth curve after selection appears to more naturally reflect the characteristics of the growth curve parameters, such as the eigenfunction of the (co)variance matrix of the RR coefficients, than when there are many age constraints.

## Figures and Tables

**Table 1:** Summary of the constraints applied to the indices

Condition^1)^	1	2		3			4			
Weeks after birth^2)^	0	0	128	0	66	128	0	43	87	128
Desired body weight (kg)^3)^	−2.5	−2.5	+4.6	−2.5	+2.8	+4.6	−2.5	+1.4	+3.9	+4.6

1) Condition number corresponds to the number used in the list of conditions presented in the Numerical example in MATERIALS AND METHODS. The number also indicates the total number of constraints applied for a given condition.

2) The day of birth is indicated as week 0.

3) Compared with before selection for breeding.

**Table 2:** Body weights (kg) achieved using our genomically enhanced breeding value–based point-gain index

Condition^1)^	Before selection	Constraint on total weight gain							

		1		2		3		4	
			
		Without	With	Without	With	Without	With	Without	With
Birth weight (−2.5 kg)^2)^	27.4	24.9	24.9	24.9	24.9	24.9	24.9	24.9	24.9
Body weights at									
43 wks (+1.4 kg)	301.1	300.2	304.0	301.6	303.0	302.2	302.7	302.6	302.5
66 wks (+2.8 kg)	479.8	479.2	484.2	482.0	483.5	482.7	482.7	482.9	482.0
87 wks (+3.9 kg)	599.8	599.2	604.2	603.1	604.3	603.7	604.0	603.7	603.8
128 wks (+4.6 kg)	715.8	714.7	718.3	720.4	720.4	720.4	720.3	720.4	720.2
130 wks	720.4	719.2	722.9	725.1	725.0	725.0	724.6	725.1	724.4
Desired total weight gain			347.4		342.4		339.6		337.1
Achieved total weight gain		−127.0	347.4	222.9	342.4	271.6	339.6	288.4	337.1

1) Condition number corresponds to the number used in the list of conditions presented in the Numerical example in MATERIALS AND METHODS. The number also indicates the total number of constraints applied for a given condition.

2) Values in parentheses indicate the desired change in body weight compared with before selection for breeding.

**Table 3:** Body weights (kg) achieved using our phenotypic-based point-gain index

Condition^1)^	Constraint on total weight gain								

	Before selection	1		2		3		4	
			
		Without	With	Without	With	Without	With	Without	With
Birth weight (−2.5 kg)^2)^	27.4	24.8	24.8	24.8	24.8	24.8	24.8	24.8	24.8
Body weight at									
43 wks (+1.4 kg)	301.1	325.2	321.3	352.7	308.1	308.1	308.2	302.9	302.8
66 wks (+2.8 kg)	479.8	500.5	495.3	537.8	482.8	482.8	482.8	483.7	482.4
87 wks (+3.9 kg)	599.8	605.6	600.5	643.4	599.1	598.9	598.8	604.0	604.1
128 wks (+4.6 kg)	713.7	691.0	687.7	720.4	721.1	721.1	721.1	720.7	720.4
130 wks	720.4	695.5	692.2	724.9	726.8	726.8	726.8	725.6	724.6
Desired total weight gain			347.4		342.4		339.6		337.1
Achieved total weight gain		823.5	347.4	4,369.2	342.4	337.3	339.6	264.2	337.1

1) Condition number corresponds to the number used in the list of conditions presented in the Numerical example in MATERIALS AND METHODS. The number also indicates the total number of constraints applied for a given condition.

2) Values in parentheses indicate the desired change in body weight compared with before selection for breeding.

**Table 4:** Selection intensity needed to achieve the desired body weight gain when using our genomically enhanced breeding value (GEBV) and phenotypic point-gain indices

Condition^1)^	Constraint on total weight gain							

	1		2		3		4	
			
	Without	With	Without	With	Without	With	Without	With
GEBV index	0.071	0.121	0.127	0.131	0.128	0.235	0.132	0.364
Phenotypic index	0.922	0.934	1.524	3.560	3.563	3.564	4.955	12.980

1) Condition number corresponds to the number used in the list of conditions presented in the Numerical example in MATERIALS AND METHODS. The number also indicates the total number of constraints applied for a given condition.

## Data Availability

All data generated or analyzed during this study are included in the published article.

## SUPPLEMENTARY MATERIAL

**Appendix 1.** Estimation of phenotypic random regression parameters using a Gompertz growth curve.

**Appendix 2.** Calculation of selection response using the first eigenfunction of the (co)variance matrix of phenotypic Legendre RR coefficients.
