# Supplementary material for: Genomic and phenotypic selection indices for decreased birth weight, shortening fattening period, and regulating total weight gain in beef cattle
Source: Anim Biosci. 2024 Aug 23;38(2):223–35. doi: 10.5713/ab.24.0171 (PMC11725746; doi:10.5713/ab.24.0171)
Supplement: Supplementary file 1 [file ab-24-0171-Appendix.pdf]

Appendix 1: Estimation of phenotypic random regression parameters using a Gompertz growth curve

The weekly phenotypic covariance matrix ($\boldsymbol{WP}$) from birth through to 130 weeks of age was obtained by generating random numbers based on the phenotypic parameters of the Gompertz growth curve. The phenotypic (co)variance of the parameters of the Gompertz growth curve ($V(P_{Gompertz})$) was assumed based on Takeda and Onogi [17-18]:

$$V(P_{Gompertz}) = \begin{bmatrix} \sigma_A^2 & \sigma_{AB} & \sigma_{AK} \\ \sigma_{AB} & \sigma_B^2 & \sigma_{BK} \\ \sigma_{AK} & \sigma_{BK} & \sigma_K^2 \end{bmatrix} =$$

$$\begin{bmatrix} 13456 & 24.258417 & -0.633932283 \\ 24.258417 & 0.3721 & -0.002075752866 \\ -0.633932283 & -0.002075752866 & 0.0001 \end{bmatrix}.$$

The Gompertz growth curve is described by three parameters: A, B, and K, which are asymptotic weight, growth starting point, and maturity rate, respectively.

$\boldsymbol{WP}$ was transformed as

$$\boldsymbol{WP} = \boldsymbol{\varphi H_p \varphi'},$$

where $\boldsymbol{H_p}$ is a covariance matrix of phenotypic Legendre RR coefficients, $\boldsymbol{\varphi}$ is a $(131 \times k)$ matrix,

$$\boldsymbol{\varphi} = \begin{bmatrix} \varphi_0(t_0) & \varphi_1(t_0) & \varphi_2(t_0) & \dots & \varphi_{k-1}(t_0) \\ \varphi_0(t_1) & \varphi_1(t_1) & \varphi_2(t_1) & \dots & \varphi_{k-1}(t_1) \\ \dots & \dots & \dots & \dots & \dots \\ \varphi_0(t_{130}) & \varphi_1(t_{130}) & \varphi_2(t_{130}) & \dots & \varphi_{k-1}(t_{130}) \end{bmatrix},$$

t_i is the age standardized for the ith specific time in growing process, and $\varphi_{\boldsymbol{j}}(t_i)$ is the jth order of the Legendre polynomial ($j = 0, ..., k - 1$) evaluated at age t_i standardized. $\boldsymbol{\varphi}$ is defined by Legendre polynomial functions and does not depend on the values in matrix $\boldsymbol{H_p}$. Thus, it is possible to estimate $\boldsymbol{H_p}$ as follows:

$$\boldsymbol{H_p} = \boldsymbol{\varphi}^{-1}\boldsymbol{WP}(\boldsymbol{\varphi}^{-1})'.$$

Appendix 2: Calculation of selection response using the first eigenfunction of the (co)variance matrix of phenotypic Legendre RR coefficients

The ith eigen function ($\mathbf{E}_{\mathrm{pi}}$) can be written as

$$\mathbf{E}_{\mathrm{pi}} = \boldsymbol{e}_{p-i}\boldsymbol{p}_{\boldsymbol{\alpha_L}},$$

where $\boldsymbol{e}_{p-i}$ is the phenotypic i^{th} eigen vector, and $\boldsymbol{p}_{\boldsymbol{\alpha_L}} = [\alpha_o \quad \alpha_1 \quad \cdot\cdot \quad \alpha_{k-1}]$ = phenotypic Legendre RR coefficient (order = 0, 1, …, k − 1).

Selection response to body weight at j weeks of age ($r_{j-week\ bw}$) due to the first eigenfunction of the phenotypic (co)variance matrix of the Legendre RR coefficients is described as

$$r_{j-week\ bw,} = \frac{cov\left(\boldsymbol{\varphi}'_j\boldsymbol{\alpha}_{\boldsymbol{L}},\boldsymbol{p}_{\boldsymbol{\alpha_L}}\boldsymbol{e}_{\boldsymbol{p-i}}\right)}{[\boldsymbol{e}'_{p-i}\boldsymbol{V}_{\boldsymbol{P}_{\boldsymbol{\alpha L}}}\boldsymbol{e}_{\boldsymbol{p-i}}]}\bar{\imath}\sqrt{[\boldsymbol{e}'_{\boldsymbol{p-i}}\boldsymbol{V}_{\boldsymbol{P}_{\boldsymbol{\alpha L}}}\boldsymbol{e}_{\boldsymbol{p-i}}]} = \frac{\boldsymbol{\varphi}'_j\boldsymbol{G}_{\boldsymbol{\alpha L}}\boldsymbol{e}_{\boldsymbol{p-i}}}{\sqrt{[\boldsymbol{e}'_{\boldsymbol{p-i}}\boldsymbol{V}_{\boldsymbol{P}_{\boldsymbol{\alpha L}}}\boldsymbol{e}_{\boldsymbol{p-i}}]}}\bar{\imath},$$

where $\boldsymbol{\varphi}'_j$ is a $(1 \times \mathrm{k})$ row vector of the Legendre polynomial ($j = 0, \dots, k - 1$) evaluated at j weeks of age standardized, $\boldsymbol{\alpha_L}$= a $(\mathrm{k} \times 1)$ column vector of the true genetic Legendre RR

coefficients, $\boldsymbol{V}_{\boldsymbol{P}_{\boldsymbol{\alpha L}}}$ is a (co)variance matrix of phenotypic Legendre RR coefficients, $\boldsymbol{G}_{\boldsymbol{\alpha L}}$ is a $(k \times k)$ (co)variance matrix of true genetic Legendre RR coefficients, and $\bar{\iota}$ is intensity of selection.
